# Delayed diagnosis of incomplete Kawasaki disease in a 3-month-old infant leading to giant coronary aneurysms

**DOI:** 10.1093/omcr/omag086

**Published:** 2026-06-08

**Authors:** Nadia Mebrouk, Loubna Chtouki, Bouchra Chkirate

**Affiliations:** Department of Pediatrics IV, Pediatric Cardiology Unit, Children’s Hospital of Rabat, Ibn Sina University Hospital Center, Rabat 10100, Morocco; Department of Pediatrics IV, Pediatric Cardiology Unit, Children’s Hospital of Rabat, Ibn Sina University Hospital Center, Rabat 10100, Morocco; Department of Pediatrics VI, Pediatric Rhumatology Unit, Children’s Hospital of Rabat, Ibn Sina University Hospital Center, Rabat 10100, Morocco

**Keywords:** Kawasaki disease, incomplete Kawasaki disease, infant, coronary artery aneurysm, prolonged fever

## Abstract

Kawasaki disease (KD) is an acute systemic vasculitis that and a leading cause of acquired heart disease in children. In infants younger than 6 months, incomplete forms are frequent and are associated with a higher risk of coronary complications due to delayed diagnosis. We report the case of a 3-month-old infant presenting with prolonged high-grade fever, initially treated as a severe bacterial infection. Retrospective identification of transient mucocutaneous signs, together with persistent inflammatory syndrome and extreme thrombocytosis, led to further investigations. Echocardiography revealed multiple coronary aneurysms. A diagnosis of incomplete Kawasaki disease was established. Combined treatment with intravenous immunoglobulins, corticosteroid therapy, aspirin, and anticoagulation resulted in rapid improvement. This case highlights the diagnostic challenges of incomplete KD in young infants and shows that a favorable outcome with partial regression of coronary lesions can be achieved, even in the presence of a giant coronary aneurysm, when appropriate management is instituted.

## Introduction

Kawasaki disease (KD) is an acute systemic vasculitis and one of the leading causes of acquired heart disease in children. It primarily affects medium-sized arteries, with a predilection for the coronary arteries, potentially leading to aneurysm formation [1]. In infants, the presentation is often atypical, with incomplete or transient mucocutaneous signs, making diagnosis difficult. Prolonged fever associated with marked inflammatory syndrome may mimic a severe bacterial infection and thus delay appropriate management. Such diagnostic delays increase the risk of coronary complications, particularly giant aneurysms, which are more common in young infants [2]. We report the case of a 3-month-old infant presenting with prolonged fever and atypical signs, in whom incomplete KD was diagnosed only after the appearance of severe coronary involvement, underscoring the diagnostic difficulty and the need for early recognition in this age group.

## Case report

**Figure 1:**
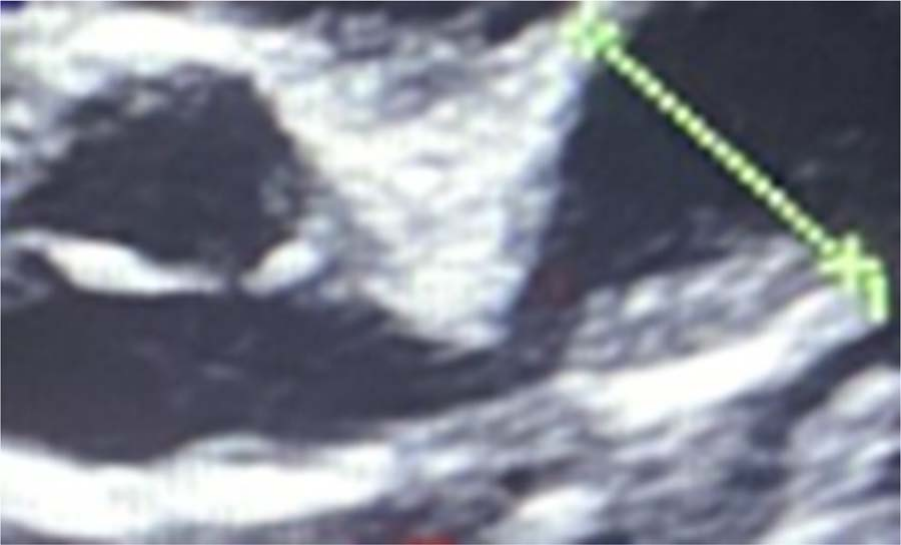
Giant left anterior descending coronary artery aneurysm with intraluminal thrombus.

A 3-month-old infant with no significant past medical history, was hospitalized for prolonged high-grade fever. The illness began with a persistent fever (39–40°C), associated with irritability, vomiting, and refusal to feed, prompting hospitalization in a peripheral hospital. Initial biological investigations revealed marked inflammatory syndrome with neutrophil-predominant leukocytosis, thrombocytosis, and elevated C-reactive protein. A severe bacterial infection was suspected, and several courses of intravenous antibiotics were administered without clinical improvement. During the illness, a transient polymorphous rash appeared. In view of the persistence of fever and worsening inflammatory markers, the patient was referred to our institution on day 22 of evolution. Retrospective history-taking revealed perineal erythema followed by desquamation. Physical examination was otherwise unremarkable apart from persistent fever. Biological investigations confirmed severe inflammatory syndrome with extreme thrombocytosis (>1 300 000/mm^3^), elevated ferritin and troponin levels, and a negative infectious workup. The diagnosis of incomplete Kawasaki disease was considered. Transthoracic echocardiography revealed multiple coronary artery aneurysms, including a giant aneurysm of the left anterior descending artery measuring 13 mm and containing an intraluminal thrombus (Fig. 1). Coronary computed tomography-angiography confirmed these abnormalities and demonstrated extra coronary arterial involvement with a small aneurysm of the left subclavian artery.

The patient received:

- intravenous immunoglobulins (2 g/kg);
- corticosteroid therapy (intravenous methylprednisolone pulses followed by oral tapering therapy);
- low dose aspirin;
- initial anticoagulation with low-molecular-weight heparin.

This therapeutic approach was consistent with current American Heart Association recommendations for high-risk forms of Kawasaki disease with severe coronary involvement [1].

**Figure 2:**
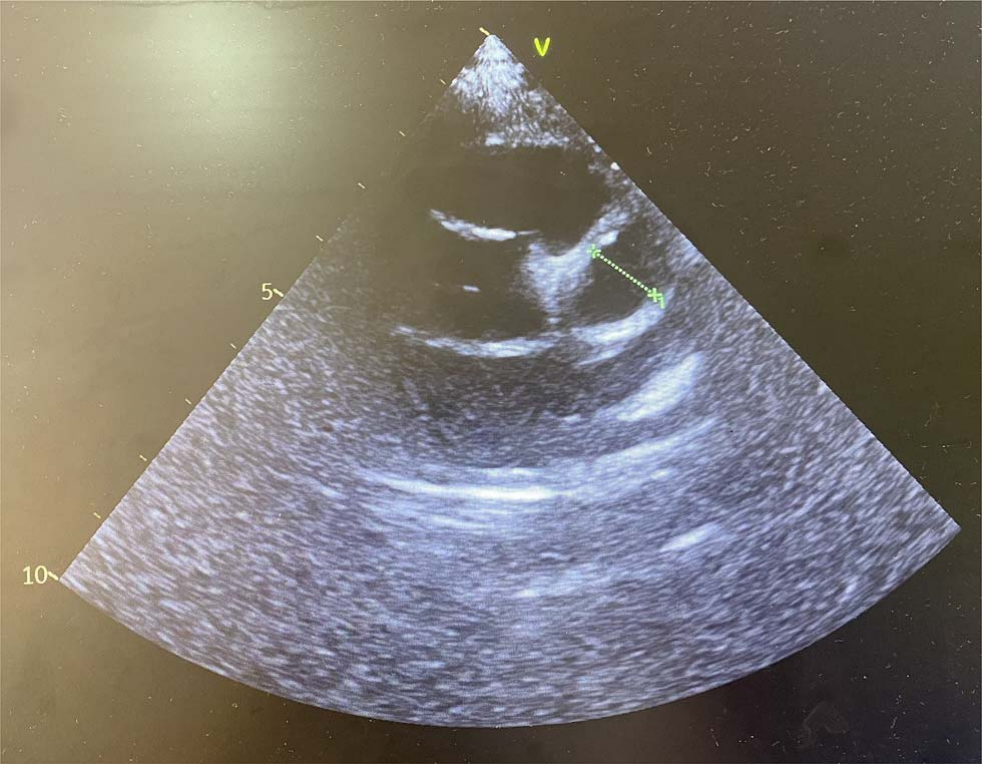
Residual mural thrombosis after giant coronary aneurysm.

The clinical course was favorable, with rapid disappearance of fever and improvement of inflammatory markers. At the 1-month echocardiographic follow-up, the following were noted:

- regression of the intraluminal thrombus to a residual mural thrombus and reduction of the left anterior descending artery aneurysm from 13 mm to 8 mm (Fig. 2);
- regression of the other coronary abnormalities.

However, the left anterior descending artery aneurysm remained classified as a giant coronary aneurysm (≥8 mm).

Due to the persistence of a giant coronary aneurysm, prolonged antithrombotic therapy was initiated, consisting of:

- low dose aspirin;
- oral anticoagulation with a vitamin K antagonist (target INR between 2 and 3).

Close cardiological follow-up was established. Long-term cardiological follow-up was established, based on regular echocardiographic surveillance supplemented, when necessary, by advanced imaging to assess ischemic risk.

## Discussion

Kawasaki disease (KD) is an acute systemic vasculitis of medium-sized arteries and remains the leading cause of acquired heart disease in children. Infants younger than 6 months represent a particularly high-risk group because they frequently present with incomplete or atypical forms and have a significantly higher incidence of coronary abnormalities [1, 3]. In this age group, prolonged fever may be the only initial manifestation, often mimicking a severe bacterial infection. This frequently leads to delayed diagnosis and treatment, a well-recognized risk factor for the development of coronary aneurysms, particularly giant aneurysms [2, 4]. In our case, the absence of persistent mucocutaneous signs and the predominance of inflammatory biological abnormalities initially led clinicians toward an infectious etiology.

Giant coronary aneurysms (≥8 mm) are associated with a high risk of thrombosis, myocardial infarction, and sudden death. Several reports have described fatal outcomes in infants with incomplete Kawasaki disease complicated by giant coronary aneurysms [5, 6]. The presence of an intraluminal thrombus further increases the risk of myocardial ischemia and sudden death [7]. This thrombotic risk is particularly high in the context of marked inflammation and extreme thrombocytosis, as observed in our patient.

The major role of intravenous immunoglobulins in reducing coronary complications has been demonstrated in landmark studies showing a significant decrease in the formation of coronary aneurysms when they are administered early in the course of the disease [8]. According to American Heart Association recommendations, administration of intravenous immunoglobulins within the first 10 days of illness significantly reduces the risk of coronary aneurysms [1]. In contrast, delayed treatment is associated with more severe coronary involvement, particularly the occurrence of giant aneurysms.

Unlike many previously reported fatal cases, our patient survived despite the presence of a giant 13 mm aneurysm of the left anterior descending artery with intraluminal thrombus. Favorable outcomes with partial regression have been reported when aggressive combined treatments are instituted rapidly, even in patients with advanced coronary involvement [9]. In our observation, although administration of intravenous immunoglobulins was delayed until day 22 of evolution, the rapid initiation of intensive combined treatment after diagnostic confirmation contributed to clinical improvement and partial regression of the coronary lesions.

In high-risk patients, particularly young infants or those already presenting with coronary involvement, adjunctive corticosteroid therapy is recommended. The rapid regression of the intraluminal thrombus observed in our patient also highlights the importance of early and aggressive antithrombotic management. Recent studies have also highlighted the growing role of advanced imaging and intensive antithrombotic strategies in improving the prognosis of these high-risk patients.

This case highlights three major clinical implications. First, incomplete Kawasaki disease should be considered in any infant presenting with unexplained prolonged fever and marked inflammatory syndrome, even in the absence of persistent mucocutaneous signs. Second, early echocardiographic evaluation is crucial in infants with persistent inflammation, as coronary involvement may already be present at the time of diagnosis. Third, a favorable outcome with partial coronary regression is possible even in forms complicated by a giant coronary aneurysm, provided that rapid multidisciplinary management is instituted. Long-term cardiological follow-up remains essential because patients with giant aneurysms remain at persistent risk of thrombosis, stenosis, and ischemic events.

## Conclusion

This case highlights the diagnostic difficulty of incomplete Kawasaki disease in young infants, whose presentation is often dominated by prolonged fever initially considered to be of infectious origin. The persistence of severe inflammatory syndrome associated with thrombocytosis should prompt early consideration of Kawasaki disease, even in the absence of complete clinical criteria. Early echocardiographic evaluation is essential to detect coronary involvement, initiate treatment promptly, and prevent severe complications. Long-term cardiological follow-up is essential in these patients.

## Consent

Oral informed consent was obtained from the patient’s parents for the publication of this case report.

The study did not require approval by the Ethics Committee of our university.
